# Hybrid high-intensity interval training using functional electrical stimulation leg cycling and arm ski ergometer for people with spinal cord injuries: a feasibility study

**DOI:** 10.1186/s40814-022-00997-2

**Published:** 2022-02-22

**Authors:** M. Vestergaard, K. Jensen, B. Juul-Kristensen

**Affiliations:** 1 The Specialized Hospital for Polio and Accident Victims, Roedovre, Denmark; 2 Department of Sports Science and Clinical Biomechanics, University of Southern Denmark, Odense, Denmark

**Keywords:** Paraplegia, Adverse events, Shoulder pain, Training intensity, Compliance, VO_2_peak, Peak watts

## Abstract

**Aim:**

The aim was to assess safety and feasibility of Hybrid High-Intensity Interval Training (HIIT) using Functional Electrical Stimulation (FES) leg cycling and arm ski ergometer in people with Spinal Cord Injuries (SCI).

**Method:**

Eight outpatients (mean age 42.8 years; 7 men) with stable SCI paraplegia (mean 14.5 years since injury) participated in hybrid HIIT (90% peak watts; 4 × 4–min intervals), three times a week (over 8 weeks). Primary outcomes were Adverse Events (AE), participant acceptability, shoulder pain, training intensity (% peak watts), and attendance.

Secondary outcomes were effect on peak oxygen uptake (VO_2_peak) during FES hybrid poling, mean watts, self-reported leisure time physical activity, quality of life, and fatigue.

**Results:**

No serious AE occurred; acceptability with the training modality was high, while shoulder pain increased by 9% (*SD* 95.2). During training, 50% of the participants reached > 90% peak watts during the intervals, three with the legs (FES cycle) and one with the arms (Ski-Erg). Overall, mean training intensity (% peak watts) was 92% (*SD* 18.9) for legs and 82% (*SD* 10.3) for arms. Proportion of fulfilled training minutes was 82% (range 36–100%); one participant dropped out after 6 weeks due to back pain. Mean VO_2_peak increased by 17% (*SD* 17.5). Participants reported increased leisure time physical activity and health-related quality of life, besides reduced fatigue.

**Conclusion:**

Hybrid HIIT was safe for people with SCI paraplegia. The majority of the criteria for feasibility were met with acceptable attendance rate, limited drop out, participants enjoyed training, and increased VO_2_peak and mean watts. However, the intensity of 90% peak watts was reached by < 60% of the participants despite high RPE ratings during training. The method of measuring and calculating intensity needs to be studied further before a study using this HIIT protocol is undertaken.

**Trial registration:**

Clinicaltrials.gov, NCT04211311, registered 12 December 2019 retrospectively registered

**Supplementary Information:**

The online version contains supplementary material available at 10.1186/s40814-022-00997-2.

## Introduction

Mortality in people with Spinal Cord Injury (SCI) is higher than in the general population, with cardiovascular disease (CVD) being one of the leading causes of early death [1–4]. People with SCI have high risk factors for CVD (overweight, hypertension, dyslipidemia, and diabetes mellitus) [5]. Neuromuscular changes following SCI reduce the ability to benefit from exercise [6], and furthermore, people with SCI have the lowest level of physical activity compared with other inactive populations [7].

It is well-known that physical activity is considered the primary means for reducing risk of CVD [8–10], and recent physical activity guidelines for cardiometabolic health in SCI include recommendations of at least 30 min of moderate to vigorous intensity aerobic exercise at least three times per week [11]. Physical activity can be expressed as cardiorespiratory fitness (CRF) and measured using peak oxygen uptake (VO_2_peak). High VO_2_peak is reported to be associated with lower risk of all-cause mortality and CVD among several patient groups [12–14], with some evidence even suggesting a proportional relationship between the size of VO_2_peak and risk reduction [8].

High-intensity interval training (HIIT) is characterized as intermittent exercise with bursts of high intervals interspersed with periods of rest or low-intensity exercise. Many different protocols are used for HIIT exercise with different intensity, duration, and number of intervals performed, from short duration (30-s intervals) at very high (“all out”) intensity to 4 × 4–min HIIT (4 intervals of 4min each between 90 and 95% peak heart rate) [15–17]. All have been shown to be superior- to moderate-intensity training and a powerful stimulus to elicit improvements in mitochondrial content and VO_2_max [18]. The 4 × 4 HIIT is recommended to induce the biggest changes in VO_2_peak [19], and HIIT has shown superior effect on cardiovascular function compared with moderate-intensity continuous training (MICT) in people with coronary heart disease, heart failure, hypertension, metabolic syndrome, lifestyle-induced chronic disease, in post-menopausal women and obese adults [19–22]. For people with SCI, only a few studies have been conducted on the effect of HIIT, primarily with arm-only exercise modalities [23–27]; however, arm-only exercise generally elicits lower VO_2_peak than leg exercise in the able-bodied [28], due to the smaller muscle mass in the arms. In people with SCI who have complete or partial paralysis in the legs, functional electrical stimulation (FES) is a way to activate the leg muscles. Metabolic rate and cardiorespiratory response were reported to be higher in hybrid leg cycling compared with arm cycling alone in people with SCI [29]. Further, a review on moderate-intensity training has shown VO_2_peak of 1.05 l/min (= 14.3 ml/kg/min) during FES leg cycling, but even higher oxygen uptake of 1.78 l/min (= 26.5 ml/kg/min) and 1.98 l/min (= 24.1 ml/kg/min) during hybrid exercise: FES cycling with arm cranking or FES rowing, respectively [30]. Only three small and uncontrolled studies [31–33] have been reported on people with SCI performing hybrid HIIT (FES leg cycling with arm cycling or rowing). One study [31] used 4 × 4–min intervals and a training period of 8 weeks. All three studies showed promising results, as hybrid HIIT was feasible and with no adverse events (AEs). Even though FES is considered both expensive and time-consuming, it has numerous advantages including augmented cardiorespiratory fitness with reduced inflammatory markers (CRP, IL-6, and TNF-α), promotion of leg blood circulation, and increased activity of specific metabolic enzymes or hormones with improvements in blood glucose control. Furthermore, it has produced greater muscle volume and fiber size, enhanced functional exercise capacity, which covers work rate, torque, speed, training time, and endurance, as well as altered bone mineral density [34–36]. With FES leg cycling combined with voluntary arm work at high intensity, it may be possible to induce a reduction in the high risk of cardiovascular disease in this population.

Besides an increased risk of CVD, people with SCI have high prevalence of shoulder pain [37], and shoulder muscle imbalance may play a role in this [38]. To reduce this shoulder muscle imbalance, a ski ergometer (SkiErg), which activates muscles on the posterior side of the upper body and arms [39], is considered a highly relevant exercise modality [40]. As hybrid HIIT in the form of FES leg cycling and arm exercise with a ski ergometer has not previously been studied in people with SCI, the effect on oxygen uptake is still unknown. Therefore, before conducting an effect study, it is relevant to investigate feasibility of this exercise modality.

The primary aim of this study was to examine safety and feasibility of the hybrid HIIT protocol, in the form of FES leg cycling combined with arm ski ergometer*,* for people with SCI paraplegia, before conducting a randomized controlled trial examining the effect of hybrid HIIT including the effect on VO_2_peak, fatigue, shoulder pain, and health-related quality of life.

## Methods

### Study design

A non-randomized pre–post design to assess the feasibility was used. All participants performed hybrid HIIT three times a week. At baseline and after 8 weeks of HIIT, the participants filled out questionnaires on shoulder pain, leisure time physical activity (LTPA), and health-related quality of life and were tested for VO_2_peak. At follow-up, the participants were further asked about their experience with the intervention.

The feasibility study adhered to the Declaration of Helsinki [41] and was approved by The Regional Committees on Health Research Ethics for Southern Denmark (nr. S-20170187, tillæg nr. 62121).

### Participants

People with SCI paraplegia attending regular training at an outpatient clinic (The Specialized Hospital for Polio and Accident Victims) were contacted by staff either directly or by telephone, then later interviewed and screened for eligibility according to the inclusion and exclusion criteria for FES cycling (listed below) and for high-intensity training as recommended by the American College of Sports Medicine. If any uncertainty about medical condition was detected, the hospital physician did the final screening. Information about injury level, completeness of injury, cause of and time since injury were retrieved from the electronic patient journal. Oral and written informed consents were given before inclusion.

Inclusion criteria were SCI paraplegia, > 18 years of age, complete or incomplete spinal cord lesions, traumatic or non-traumatic origin of injury, good muscle contraction possible through electrical stimulation, and willingness to train three times a week at high intensity. People with prior experience with FES cycling were preferred, in order to reach the state after muscle adaptation to electrical stimulation. However, to reach the pragmatically chosen number of participants, people without FES experience also had to be recruited. Exclusion criteria were the presence of a pacemaker, unstable fractures, cancer, heterotopic ossification, severe osteoporosis, pressure ulcers, recent surgery, dislocation or subluxation of any joint, being pregnant, severe autonomic dysreflexia, more than 45 as a total score on the Wheelchair Users Shoulder Pain Index (WUSPI) (range 0–150; 0 = no pain), and medical contraindications to high-intensity training such as heart problems, very low or high blood pressure, and severe autonomic dysreflexia.

### Equipment

**Fig. 1:**
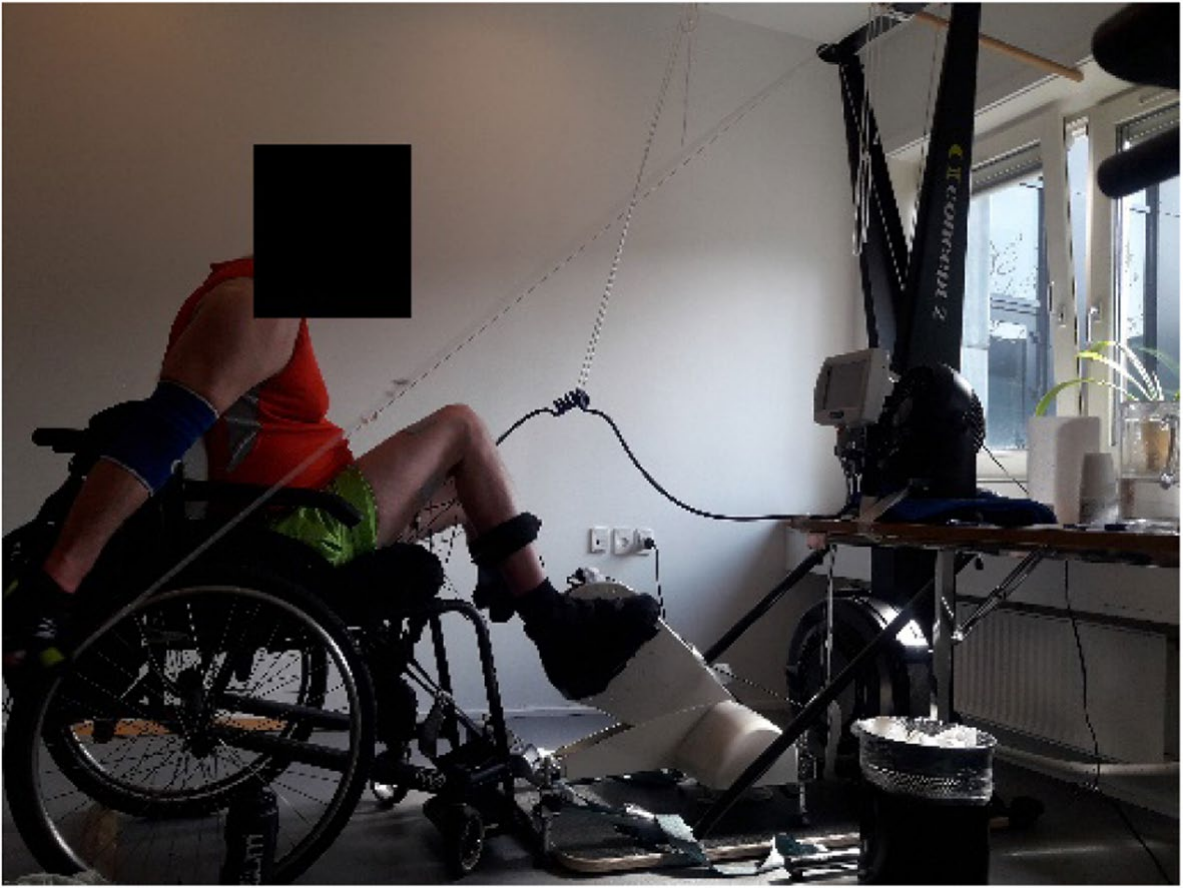
The setup with the wheelchair positioned in front of RT300 FES leg cycle while pulling the Concept2 ski ergometer. Written permission to show photograph is given by the participant

**Fig. 2:**
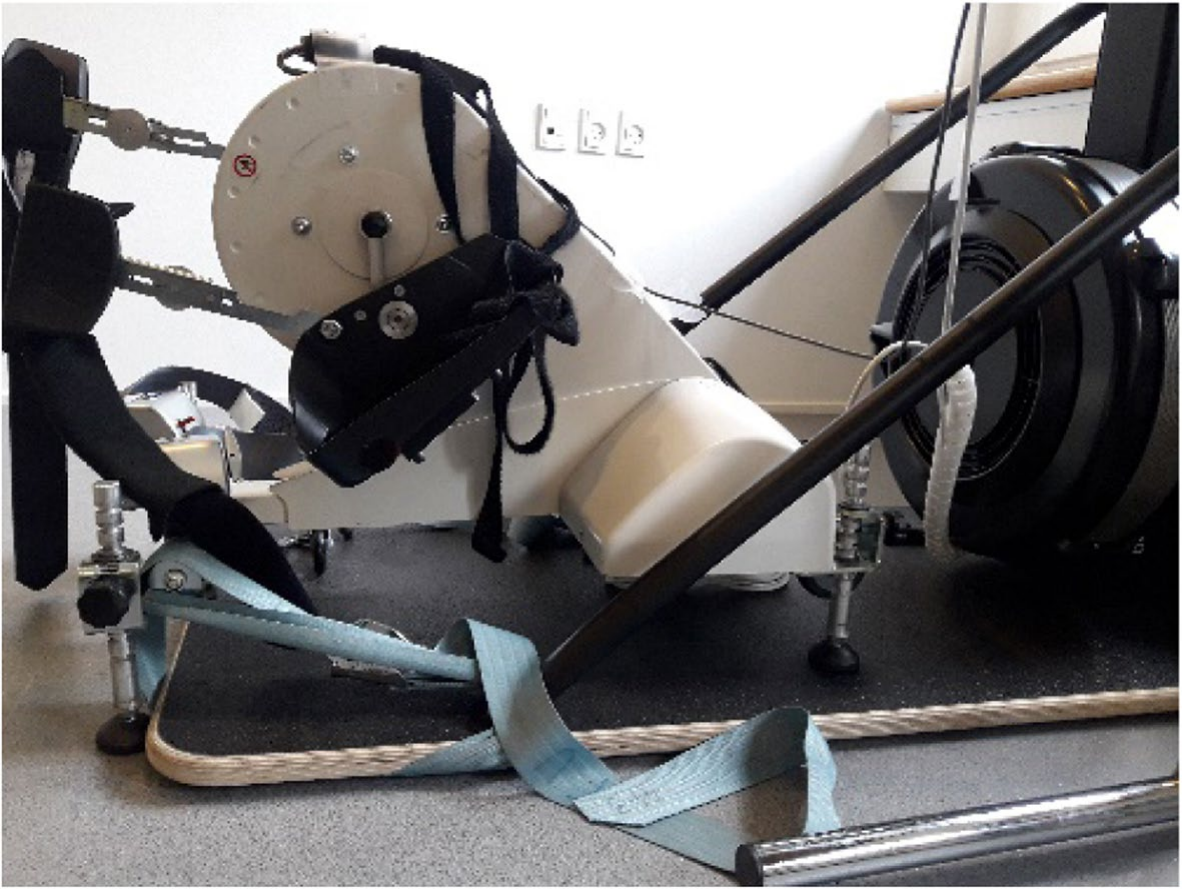
The RT300 FES leg cycle positioned as close to the Concept2 ski ergometer as possible with two legs on the ski ergometer floor plate

During the intervention and tests, all participants used their own wheelchair (Fig. 1). The FES leg cycle (RT-300 leg-cycle, Restorative Therapies, Baltimore, USA) was placed in front of the ski ergometer (Concept2 SkiErg, Morrisville, USA) with the two supporting front legs on the floor plate of the SkiErg (Fig. 2). The FES leg cycle uses a six-channel alternating monophasic, charged, and balanced waveform stimulation with pulse periods between 0 and 100 ms (default 40 ms). Self-adhesive surface electrodes (Enraf nonius en-trode 50 × 90 mm and PALS electrodes 7.5 × 10 cm) were placed bilaterally over the quadriceps, hamstrings, and gluteus maximus muscles. During a muscle test, current output threshold level per channel (0–140 mA with 100–1500 Ω load) and pulse width (50–500 μs) was set for each muscle group to elicit a good muscle contraction depending on sensation and spasticity. Speed was set to minimum of 35 RPM. The RT300 has a built-in computer that adjusts the stimulation level within the preset threshold (min–max mA). When speed is maintained, resistance can be increased manually. External leg resistance was adjusted by the principal investigator. Resistance, speed, torque, and stimulation were stored on an external server (http://www.rtidatalink.com).

For the arm exercise, the SkiErg was used, with a double pooling technique similar to classical cross-country skiing, where both handles are pulled down and backwards simultaneously. The pulling strings on the SkiErg were lengthened 67 cm to allow a full backswing with the arms. External resistance was set to 1 on the flywheel for all participants to avoid muscle fatigue. Data from the SkiErg were collected from the performance monitor (PM5) via a memory stick.

### Test protocol

VO_2_peak and peak watts were measured during incremental ramp tests to volitional fatigue and were used not only as a basis for establishing the optimal intensity level during intervention, but also as effect measures at baseline and post intervention. Three tests were performed on the same day: firstly, a test using the legs alone; secondly, a test using arms alone, aiming to establish a starting watt level for the third test; thirdly, the hybrid test which included legs and arms simultaneously. Peak oxygen uptake and peak watts were measured from the hybrid test. Watts for legs and arms were recorded separately.

Due to the various performance abilities in the group of included subjects, we had to adjust the stimulation individually to try to reach maximal stimulation within a duration of 4–8 min in test 3 (same stimulation protocol used individually in pre- and posttest).

Warm up for test 1 (legs alone) started with 2 min of passive leg cycling, followed by 8 min with stimulation at low resistance to reach a steady response to the stimulation and reduce possible spasticity. In increments of 30–60 s, the level of resistance was increased by 0–3 W until the watts could no longer be maintained. Leg movement was stopped, and with feet still strapped to the pedals, the participants started pulling the SkiErg, warming up for 3 min at approximately 15 W. When test 2 (arms alone) started, the level of resistance was increased by 5–20 W in increments of 30 s until watts could no longer be maintained. To avoid fatigue in the arms, participants were not pushed to an absolute maximum. Subsequently, there was 3 min of absolute rest. Thereafter, test 3 (the hybrid test) began with 1 min ramping up stimulation on the legs, where the participant started pulling the SkiErg into a steady rhythm, based on the starting watt level for arms and legs. Resistance was then increased by 5–10 W on the SkiErg, and by 0–3 watts on the FES cycle in increments of 30 s or 1 min, until the watts could no longer be maintained. If watts on the legs decreased before the arms, the test continued until neither arms nor legs could maintain the preset resistance level of watts. The different watt increase and duration of increments were required to reach a test duration of 4–8 min as recommended [42]. For the legs, peak watt was defined as an average of the three highest watt values within 30 s, while for the arms, peak watt was defined as the highest average of 1-min splits. Participants wore a mouthpiece during all three tests, measuring ventilatory parameters and pulmonary gas exchange, using a metabolic card (CPS, Innovision, Denmark), which had been calibrated pre-exercise according to manufacturer specifications. Criteria that determined when VO_2_peak was reached, was respiratory exchange ratio (RER) of 1.05 or higher, and concentration of blood lactate (7 mmol/l or more) measured in the left index finger just after completion of the hybrid test and again after 3 min, using a lactate analyzer (EKF Biosen C-line, Germany). Also, subjective rated perceived exertion (RPE) over a minimum of 16 on the Borg 6–20 scale was used as the criterion. Because measurement of VO_2_peak during the intervention was not possible, peak watts were used as an indication of maximum intensity during the intervention. Changes in power outputs during training will indicate changes in peak power output and accordingly changes in VO_2_peak [43].

### Intervention

The intensity used during HIIT long intervals is defined as the exercise intensity eliciting 90–100% of VO_2_peak (Watts at VO_2_peak) [44]. In practice, if peak watt is defined as the intensity that will elicit a 100% VO2peak within a single bout of 4–6 min, then training for the 4 times of 4-min interval session could be performed at 90% of peak watt to be sustained during the whole training session. Due to the individual stimulation protocols used to define peak watt in this study, peak watt was only used as an indicator for maximal training intensity, and focus was on having the highest possible intensity during the whole training session. The intervention consisted of 4 × 4–min hybrid intervals with 2 min active rest in between, where participants were instructed to keep their warm-up pace in order to remove buildup of lactate. Training started with a 10-min warm-up, after which the participant aimed for 90% of peak watts for arms and legs in each 4-min interval; however, as mentioned above, the most important was completion of all four intervals at the highest possible intensity. For those with motor complete lesions, the clinician controlled the intensity for the legs. Both the FES cycle and the SkiErg registered mean watts for every interval, and at the end of each training session, the mean of the obtained watts from all four intervals was registered and reported. Additionally, intensity was rated subjectively by the participants after each interval using the Borg 6–20 scale [45].

### Outcomes

Evaluation of safety and feasibility was performed using predefined criteria with pragmatic cut-points (Table 1), as previously recommended [46]. This included the following primary outcomes: AEs, participant acceptability and preference with the training and test protocol, self-reported shoulder pain, registered training intensity, and attendance. Secondary outcomes included VO_2_peak, mean watts, self-reported LTPA, quality of life, and subjective fatigue.

**Table 1:** Criteria to evaluate safety and feasibility

Outcome	Stop, do not continue	Continue	Continue with modification
Adverse events	More than 1 incidence of autonomic hyperreflexia or acute cardiac event during training	Less than or equal to 1 incidence of autonomic hyperreflexia or acute cardiac event during training	
Participant acceptability	More than 50% of the participants rated experience during and after training higher than 3 (on a 1–7–point Likert scale, 1 = most acceptable)	Less than or equal to 50% of the participants rated experience during and after training higher than 3 (on a 1–7–point Likert scale, 1 = most acceptable)	
Participants preference for training	More than 50% of the participants preferred continuous training to interval training	Less than or equal to 50% of the participants preferred continuous training to interval training	
Shoulder pain	Individual WUSPI PC score ≥ 45 after the intervention	Individual WUSPI PC score < 45 after the intervention	
Intensity			Less than 60% of participants reached the desired intensity of 90% peak watts
Attendance			Less than 60% of total training minutes were fulfilled.

Evaluation criteria, based on recommendations from Thabane, were set using a three-way system: stop, do not continue, continue without modifications, continue with modifications. The criteria were set pragmatically.

#### Primary outcomes

AE included incidents of autonomic hyperreflexia, characterized as increase in systolic blood pressure greater than 20–30 mmHg accompanied by severe headache, feeling of anxiety, profuse sweating above level of injury, flushing, and piloerection (goose bumps) above the injury, besides dry and pale skin below the level of injury [47]. If participants reported any of the above symptoms, blood pressure was immediately measured (Kivex UA-787, Denmark). AE also included acute cardiac event characterized by dizziness, syncope, shortness of breath, and chest discomfort during exercise [48], and therefore, heart rate was continuously measured during the test and training using a heart rate monitor (Polar, RC3, Finland). In addition, other unintended effects (e.g., pain, spasticity, muscle soreness, and effect on sleep) were registered.

Participant experience was measured after the 8-week intervention, using a customized questionnaire based upon the Physical Activity Enjoyment Scale (PACES) [49]. Using a seven-point Likert scale (lower scores indicating higher enjoyment), participants were asked (1) how they felt during training (enjoyed/hated it, fun/boring) and (2) how they felt after training (good/awful). Further, they were asked whether (3) they preferred training with arms and legs simultaneously/only with legs/only with arms and whether (4) they preferred continuous training or interval training. Lastly, they were asked about (5) the intensity level (on a five-point verbal scale, from much too low to much too high) and (6) whether they preferred training as normal or as in the study, and with open questions (7) “How did you experience training?” and (8) “What was good and bad?” (Additional file 1).

Shoulder pain was measured using the Wheelchair Users Shoulder Pain Index (WUSPI) [50], with high test–retest reliability [51], using the Danish translated and cross-culturally validated version for spinal cord injured people [52]. It is measured via 15 items measuring shoulder pain during daily activities, with each item scored on a 10-cm visual analogue scale (range 0–150; from “no pain” (0 cm) to “worst pain ever experienced” (10 cm)) [50]. Within each item, there is a “not applicable” option, used to calculate Performance Corrected (PC) WUSPI score, dividing the total score by the number of item responses and multiplied by 15. A WUSPI score of a maximum of 45 was arbitrarily selected as the cut-point in the criteria for acceptable feasibility and safety of the study outcomes.

Shoulder/arm/hand pain before and after each training session was further measured using a numeric rating scale (NRS) 0–10 (0 = no pain, 10 = worst pain) [53].

Training intensity was based upon peak power measured during the hybrid test at baseline, with separate measures from the FES cycle and the SkiErg. Based on these measures of peak power, the aimed intensity of 90% peak watts was calculated. After approximately 11 sessions, a peak watt test was performed to adjust the training intensity to reflect the expected progression in power output (protocol available from authors upon request).

Attendance was measured as the proportion of fulfilled training minutes, with reasons for cancellation registered, as well as total dropout.

#### Secondary outcomes

Measurements of VO_2_peak and peak watts were performed as described in the test protocol (see section above).

Self-reported physical activity was measured with the SCI Leisure Time Physical Activity Questionnaire (SCI-LTPAQ) [54], with significant test–retest reliability [55]. The Questionnaire asks for the number of minutes spent performing mild-, moderate-, and high-intensity LTPAs daily. The scale is scored by multiplying the number of days of each activity level by the number of minutes, yielding the total number of minutes of activity performed during the past week.

Self-reported health-related quality of life (HrQoL) was measured using the Short Form-36 (SF-36) [56], a generic instrument with 36 questions distributed across two main categories: physical component summary (PCS: Physical Functioning, Role-Physical, Bodily Pain, General Health) and mental component summary (MCS: Vitality, Social Functioning, Role-Emotional, Mental Health). Scoring is categorically based on a Likert scale ranging from three to six categories. Scoring is calculated using an algorithm transforming scores into a 0–100 scoring system, with higher scores indicating better health [57].

Subjective fatigue was measured using the Multi-Dimensional Fatigue Inventory (MFI-20) [58]. This instrument was originally developed for cancer patients but has been tested and found to be valid and reliable also in other population groups [59]. It covers five dimensions: General Fatigue, Physical Fatigue, Mental Fatigue, Reduced Motivation, and Reduced Activity. Each item uses a five-level scale. Subscale scores (range 4–20) are calculated as the sum of item ratings, and a total fatigue score (range 20–100) is calculated as the sum of all subscale scores. High scores indicate higher levels of fatigue.

### Data analysis

Based on the current feasibility study design, a sample size calculation for this study and a future RCT were not performed, as previously recommended [46]. Since no prior study has reported data on this training modality, a pragmatically chosen sample size of eight participants was selected (Table 2). As the nature of this feasibility study does not support statistical analysis of the primary outcomes, data are presented descriptively for each individual and for continuous data as group mean (SD)/median (range).

**Table 2:** Participant characteristics

Patient	Age (years)	Gender (m/f)	Level of injury	Years since injury (years)	Motor completeness of injury	Weight baseline (kg)	Height (cm)	FES cycled before
1	66	m	L1	4	Incomplete	94.4	184	Yes
2^a^	34	m	TH5	15	Incomplete	95.2	183	Yes
3	20	m	TH8	3	Complete	85.9	183	Yes
4	40	m	TH7	13	Complete	91	202	Yes
5	29	m	TH10	1	Incomplete	66.4	188	No
6	57	m	TH8	29	Complete	99.4	200	Yes
7	50	m	L2	5	Incomplete	114.2	186	No
8	46	f	TH4	46	Incomplete	77.5	165	Yes
Mean	42.75			14.5		90.5	186.4	

^a^Indicates the participant who dropped out after 6 weeks

## Results

**Fig. 3:**
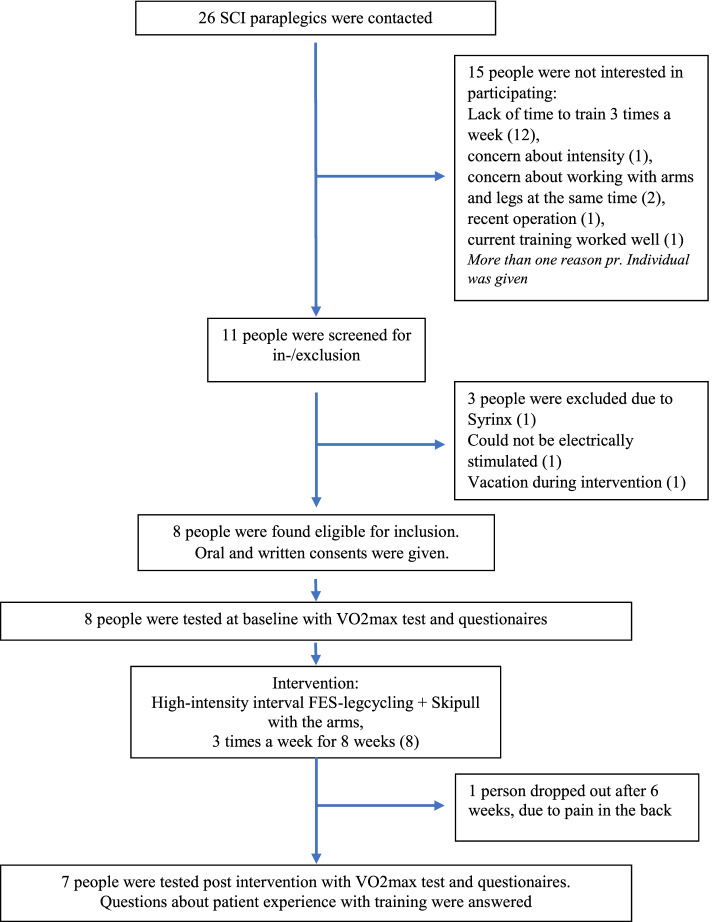
Flowchart of how the 8 eligible participants were found out of the 26 persons who were contacted, with reason for not wanting to participate (15) and reasons for exclusion (3) presented

Enrollment took place in January and February 2018. Twenty-six people were contacted, of which 11 were interested in participating, while three had to be excluded (Fig. 3 flowchart).

Recruitment rate was 31%, with a dropout rate of 12.5%. Mean age of participants was 42.8 years (*SD* 15.11), with a majority being men (87.5 %), with an even distribution in complete and incomplete SCI injuries. All participants used a manual wheelchair for primary transportation, only one had standing function, and four were active in their leisure time.

### Primary outcomes

No episodes of serious AEs occurred. Two participants experienced mild headache after training, but showed no other signs of autonomic hyperreflexia. Only a few minor adverse effects occurred: slight non-persisting pain in neck (*n* = 1), arms and shoulders (*n* = 4) during and between training sessions, dizziness that disappeared after 5 min (*n* = 1), feeling tired in the head/dizziness that disappeared after training with no other signs of autonomic hyperreflexia (*n* = 2), increased spasms (*n* = 2), and vomiting just after training (*n* = 2).

All participants reported a positive experience with the training. Comments were the following: “*Formidably*, *extremely good, very positive experience, really good, great to get the pulse up and to sweat, good to feel you had worked hard sitting*”. “*Training was hard but good. The good thing was that there was someone encouraging and forcing you to get the training done*”. “*It was fun trying it, but I would not miss my other training in the different machines, training different muscle groups, and it is important for me to talk to the other people in the gym*”. “*I liked the intensive training, but the time spent applying the electrodes made me impatient”. “Bad that the training ended”.*

No individual WUSPI PC score exceeded 45 points post intervention. There was a slight overall increase in shoulder pain of 9% in the WUSPI PC score, with a large variation from a 97% decrease to a 204% increase (Table 3).

**Table 3:** Primary outcomes of WUSPI (shoulder scores), training intensity, and attendance rate (*n* = 7).

ID no.	1	3	4	5	6	7	8	Mean
WUSPI, pre intervention (PC score)	10.1	39.2	9.9	-	15.2	1.1	0	11
WUSPI, post intervention (PC score)	32	27	0.3	1.8	22.9	0.7	0.8	12
WUSPI PC change (%)	204	− 32	− 97	0	50	− 33	1	9
Intensity FES cycle % peak watts (*SD*)	87 (*28.1*)	103.9 (*40.8*)	87.5 (*9.7*)	70.2 (*11.8*)	92 (*20.1*)	127 (*29.4*)	76.3 (*4.4*)	92 (*18.9*)
Intensity SkiErg % peak watts (*SD*)	103 (*25.8*)	71.4 (*22.7*)	76 (*3.6*)	77 (*2.1*)	85 (*3.8*)	79 (*7.6*)	80 (*6.2*)	82 (*10.3*)
Attendance (% fulfilled training minutes)	100	65	91	36	100	83	100	82

WUSPI-PC (Wheelchair Users Shoulder Pain Index–Performance Calculated): total score divided by number of items multiplied by 15 (higher score = higher pain). Intensity is the average of achieved watts during intervals throughout the training period as proportion of peak watt from the baseline test.

Mean intensity was 92% (*SD* 18.9) on the FES leg cycle, and 82% (*SD* 10.3) on the SkiErg (Table 3), but the aimed intensity of 90% peak watts was reached by only 43% (three participants) on the FES leg cycle and by 14% (one participant) on the SkiErg. Group mean RPE during the 8-week training period, measured after each interval was 17 (*SD* 1.35).

Attendance was 82% with large variation (36 to 100% completion). Reasons for not attending training sessions were not directly related to the training (influenza, urinary tract infection, hospital and local council appointments, problems with transportation, personal and family matters, oversleeping, lack of sleep due to neurogenic pain, cancellation without reason).

The majority of the feasibility criteria were fulfilled, except for the intensity, which has to be modified in a future effect study (Table 4).

**Table 4:** Outcomes in the feasibility progression criteria (evaluating either stop or continue with modification) (*n* = 7 as one person dropped out due to back problems at Week 6).

Criteria	Result (***n***/%)	Evaluation
Adverse events		
Number of incidents of autonomic hyperreflexia	0	Continue
Number of incidents of acute cardiac events	0	Continue
Participant acceptability		
Number of participants who rated experience during and after training higher than 3 (on a 1–7–point Likert scale with one being most acceptable)	1	Continue
Participant preference for training		
Number of participants preferring continuous training to interval training	1	Continue
Shoulder pain		
Number of participants with WUSPI PC score > 45 after the intervention	0	Continue
Intensity		
Proportion of participants who reached 90% peak watts		
FES leg cycle	43%	Continue with modification
SkiErg	14%	Continue with modification
Attendance		
Proportion of fulfilled training minutes (total per person = 805 min)	82%	Continue

### Secondary outcomes

Mean VO_2_peak (pre to post) increased from 1.64 l/min^-1^ (*SD* 0.39) to 1.91 l/min^-1^ (*SD* 0.61), equal to 17% (*SD* 17.47) increase (Table 5).

**Table 5:** Secondary outcomes of VO_2_peak, RER, HRpeak, and peak watts during hybrid test (from pre to post intervention; *n* = 7). Data are presented as mean (SD).

Variables	Pre intervention	Post intervention
VO_2_peak l/min^-1^	1.64 (0.39)	1.91 (0.61)
VO_2_peak ml/kg/min	18.25 (2.82)	21.21 (5.62)
RER	1.17 (0.08)	1.15 (0.06)
HRpeak	155 (28)	166 (35)
FES peak watts	18.57 (26.78)	19.57 (27.50)
SkiErg peak watts	54.71 (22.25)	76.21 (17.30)

*VO*_*2*_*peak* peak oxygen consumption, *RER* respiratory exchange ratio, *HRpeak* peak heart rate, *FES* functional electrical stimulation leg cycling

Mean watts during training sessions (first to last training session) increased 7% (*SD* 0.69) on the FES cycle and 17% (*SD* 5.67) on the SkiErg.

There was an overall increase in self-reported LTPA of 408% for light and 700% for hard LTPA. For health-related quality of life (SF-36), there was an increase in PCS (10%), and in MCS (7.5%). Overall, there was a decrease in fatigue (MFI-20) ranging from 15% (for general fatigue) to 42% (for reduced activity) (Table 6).

**Table 6:** Secondary outcomes of self-reported LTPAQ-SCI, SF-36, and MFI-20. Data are presented as mean (SD) and change (%)

Variables	Pre intervention (*n* = 6) Mean (SD)	Post intervention (*n* = 6) Mean (SD)	Absolute difference (%)
LTPAQ-SCI, min per week			
Light LTPA,	123 (139.24)	625 (668.67)	502 (408%)
Moderate LTPA	670 (826.22)	355 (544.12)	315 (47%)
Hard LTPA	20 (44.72)	160 (137.11)	40 (700%)
SF-36 (higher score = higher QOL)			
PCS	39.22 (6.39)	43.04 (9.05)	3.82 (10%)
MCS	47.42 (11.33)	50.99 (7.06)	3.57 (8 %)
MFI-20 (lower score = lower fatigue)			
General fatigue	54.17 (30.33)	45.83 (25.94)	− 8.34 (15%)
Physcial fatigue	44.79 (18.19)	33.33 (18.28)	− 11.46 (26%)
Reduced activity	32.29 (20.23)	18.75 (19.76)	− 13.54 (42%)
Reduced motivation	34.38 (16.04)	22.92 (13.82)	− 11.46 (33%)
Mental fatigue	31.25 (22.10)	25 (22.01)	− 6.25 (20%)

*LTPAQ-SCI* Leisure Time Physical Activity Questionnaire-SCI, *SF-36* Short Form 36 version 2, *MFI-20* Multidimensional Fatigue Inventory

## Discussion

No serious AEs occurred; all participants gave positive feedback, with small increases in shoulder pain. Training intensity of 90% peak watts was reached by less than 60% of the participants for FES leg cycling and SkiErg. Most participants were compliant, with one dropout after 6 weeks due to back pain. Mean VO_2_peak increased by a mean of 17%, and mean watts by 7% on the FES leg cycle and 17% on the SkiErg. Participants reported increased LTPA, health-related quality of life, besides reduced fatigue.

No serious adverse events occurred during the current hybrid HIIT training, despite two participants with their lesion level above T6 and one participant aged 66. This is in line with other studies of high-intensity training for SCI [24, 25, 31], and with a systematic review of cardiovascular training for people with SCI [60], where no serious adverse events were reported. Generally, high-intensity cardiovascular training is considered safe, as moderate- to high-intensity is part of the recommendations for SCI populations [6, 11], as well as groups with other diagnoses [13, 19–21, 61], and for the general population to induce a positive effect on health [10, 16, 62–64]. In SCI, the health benefits of high-intensity training are reported to overweigh its potential negative risks [60]. A prerequisite, though, is that participants are pre-screened for cardiac health risks and autonomic hyperreflexia [60].

The current participants reported that it felt good to be out of breath, and that it gave them a feeling of working hard, a feeling they could not accomplish during their regular training. The current positive feedback is in line with a previous study reporting higher enjoyment with high-intensity training than with moderate-intensity training [65]. The current positive participant experience is essential for using the current training protocol in a future effect study since high-intensity training requires high motivation.

No participant exceeded a WUSPI PC score of 45 post intervention, but surprisingly, there was a slight overall increase in shoulder pain. This was due to a large increase in only one participant, who surprisingly had rated shoulder pain 0 (0–10 NRS) after all training sessions. The small increase in present shoulder pain is in contrast with previous findings of reduced shoulder pain following exercise programs [66], and no increase in shoulder pain using the SkiErg at high intensity [67]. The SkiErg activates the abdominal muscles and the posterior side of the trunk and arm muscles, thus potentially decreasing shoulder muscle imbalance which can cause shoulder pain. It may also potentially improve sitting posture and balance [68].

The current mean intensity of 92% on the FES leg cycle and 83% on the SkiErg almost corresponds with the aimed intensity of 85–95% achieved in a previous small study combining FES leg cycling with arm cycling [31]. Despite the higher intensity reached on the FES leg cycle than the SkiErg (92% vs 83%), improvements on the SkiErg were higher compared with the FES leg cycle (17% vs 7%). This was expected as power output during FES leg cycling is known to be low [69], since the neurological muscle impairments affected by the SCI will lead to reduced training response compared with the non-affected arms.

Two participants reached an intensity of more than 100% (103% SkiErg and 127% FES cycle). This can be explained if the participants did not reach their actual maximum in the pretest. They were not used to pushing themselves as hard as they had to in the test. The participant who reached 127% on the FES cycle had the highest level of motor function, which made it difficult to adjust the stimulation optimally and to coordinate the two movements (leg cycling and arm pulling).

Our criterion for evaluating intensity (≥ 60% of the participants had to reach 90% peak watts) was not met. The low number of participants reaching high intensity in the current study does not correspond with the majority of the participants frequently rating 18–20 on the Borg 6–20 scale at the last two intervals, along with the observed heavy breathing, and two participants vomiting due to the high intensity. This discrepancy may be due to the method of measuring and calculating intensity. Incremental ramp test is found valid to test VO_2_peak using the SkiErg [70]. However, using peak power as an outcome requires a more standardized SkiErg test protocol than used in the current study, since shorter- and higher-step increments will result in higher peak power outputs than longer- and smaller-step increments [71]. The current study was forced to use a more pragmatic and flexible test protocol due to the different abilities of the participants. In practice, this meant that the test protocol had to vary from 5-W increase per minute to 10-W increase per 30 s. For those with higher- and shorter-step increments, this may have resulted in too-high peak power measured during the test, with consequently too-high training intensity. Therefore, future studies on similar patients need either to standardize the test protocol with same step increments when using peak power as an objective outcome, or to only use subjective ratings of the intensity, thus excluding peak power as an outcome. FES cycling produces low power output [69], and it can be questioned whether it is adequate for incremental testing [30]. However, since hybrid training seems to be more effective in increasing VO_2_peak than arm work alone [30, 72], inclusion of the FES cycle in the test is relevant. Of note is that the combination of SkiErg and FES cycle has not been studied before, why validity and reliability of this hybrid peak test is unknown and need to be studied further before a study of the effect on VO_2_peak. Further, the three tests (legs alone, arms alone, both arms and legs together) were performed on the same day which most likely has influenced the result as fatigue may have occurred. In a future study, the three tests should be performed on separate days.

The 4 × 4–min interval training protocol has shown good results in other studies [19],and shorter intervals with higher intensity may neither be possible nor safe in this population. With a more standardized test protocol (same step increment), the intensity of 90% peak watts may have been feasible. Alternatively, with the interval duration maintained, the intensity may be lowered to about 75–85% of peak watts. Also, the active rest period could be prolonged to 3 min. Since there is no consensus about the optimal HIIT protocol for non-disabled people [17], it is also unknown which modifications are needed in SCI populations to induce a cardiovascular response high enough to reduce the risk of cardiovascular disease.

In studies of able-bodied people, improvements of 3.5 ml/kg/min^-1^ (1 MET) have been reported to improve survival by 10–25% [73], while in the current study, the average improvement was 2.96 ml/kg/min^-1^ in VO_2_peak, with three participants achieving improvements of 4.27, 7.83, and 6.9 ml/kg/min^-1^. Despite not meeting the criteria for intensity, an overall increase in VO_2_peak of 17% was found, suggesting that reducing the aimed intensity to 75–85% peak watts could be enough to achieve an increased VO_2_peak.

The current recruitment rate was only 31%, which is very common in heterogeneous SCI populations [74], indicating that a large number of people is required to achieve adequate power in a future RCT. Mean attendance of training minutes (total 805 min per person) was 82% varying from 39 to 100%. This low attendance rate is not in line with previous studies of HIIT in people with SCI, where 100% compliance and 100% attendance has been reported [23–25, 31]. The current attendance rate is, however, in line with clinical experience, and a review also reporting challenges in this population to adhere to exercise trials, due to transportation, mobility barriers, and secondary health problems [74].

The current dropout rate of 12.5% (one participant out of eight), is actually relatively low, compared with most other studies on SCI populations [74]. The participant who dropped out after 8 weeks due to back pain, trained with intensity of 41–69% peak watts (FES leg cycle) and 93–103% (SkiErg). This participant reported no pain or discomfort after/between each training session. In fact, this participant reported several positive effects (better sleep, better bowel function, better mood). The attendance of this participant was low (39%), but cancellation reasons were, however, unrelated to training (e.g., car would not start, hospital appointments, other illness, death in the family).

### Strengths and limitations of the study

The feasibility design per se is considered to have low methodological quality compared with an RCT, the gold standard of study designs [74]. Limitations of this study are therefore lack of a control group and no blinding. This meant that there was high potential for selection bias as only those motivated for HIIT signed up and may well have represented the most active segment of this population. Further, the small sample size made it difficult to make solid conclusions on the effect of HIIT; however, based on current and previous results, HIIT is anticipated to be safe in SCI populations. The current study aimed to assess if it was safe, possible, and relevant to train at very high intensity in this hybrid setting, but since validity and reliability of this hybrid VO_2_peak test has not been studied prior to this study, uncertainty about the calculated intensity occurs, why interpretation of intensity must be done with caution. The criteria for evaluating feasibility could only be set pragmatically from clinical experience since no prior studies on this aspect have been conducted.

One of the strengths is that this study followed the CONSORT guidelines for conducting and reporting feasibility studies [75], which increases its methodological quality. Secondly, a strength is the high relevance of estimating safety and feasibility of this training protocol, before conducting an RCT study [46]. This study makes no conclusion on the effect of hybrid HIIT on oxygen uptake, but since people with SCI have a high risk of CVD [11], it is important to conduct high-quality RCTs, investigating whether VO_2_peak can be increased sufficiently to reduce the risk of CVD in this population [6, 11, 76].

## Conclusion

The protocol was found feasible with some modifications. No serious adverse events occurred in this study, which together with the literature indicates that 8 weeks of hybrid high-intensity interval training is safe for people with SCI paraplegia. Participants enjoyed the HIIT training, with an acceptable attendance rate, and limited dropouts but less than 60% reached the aimed intensity of 90% peak watts, despite high RPE ratings during training. This indicates that more attention is needed to the method of measuring and calculating the intensity using both the SkiErg and the FES cycle simultaneously. Establishing the correct intensity is essential, before a study of the effect of hybrid HIIT on VO2peak can be performed. It is critical that medical screening for any heart condition is performed before using HIIT in this population.

## Supplementary Information

**Additional file 1.** Modified version of the Physical Activity Enjoyment Scale (PACES).

**Additional file 2.** CONSORT 2010 checklist of information to include when reporting a pilot or feasibility trial.

## Data Availability

The datasets generated and/or analyzed during the current study are available from the corresponding author upon reasonable request.
