# Supplementary material for: Hybrid high-intensity interval training using functional electrical stimulation leg cycling and arm ski ergometer for people with spinal cord injuries: a feasibility study
Source: Pilot Feasibility Stud. 2022 Feb 22;8:43. doi: 10.1186/s40814-022-00997-2 (PMC8862540; doi:10.1186/s40814-022-00997-2)
Supplement: Supplementary file 1 — Additional file 1. Modified version of the Physical Activity Enjoyment Scale (PACES). [file 40814_2022_997_MOESM1_ESM.docx]

Appendix 1. Modified version of the Physical Activity Enjoyment Scale (PACES)(1).

Questions after the intervention

How did you feel during training?

| 1 | 2 | 3 | 4 | 5 | 6 | 7 |
| --- | --- | --- | --- | --- | --- | --- |

I enjoyed it I hated it

How did you feel during training?

| 1 | 2 | 3 | 4 | 5 | 6 | 7 |
| --- | --- | --- | --- | --- | --- | --- |

I thought it was fun I thought it was boring

How did you feel after training?

| 1 | 2 | 3 | 4 | 5 | 6 | 7 |
| --- | --- | --- | --- | --- | --- | --- |

I felt good I felt terrible

- If you could choose a training method, how would you prefer to train?
- With both arms and legs
- With just legs
- With just arms

If you could choose a training intensity, how would you prefer to train?

- Continously, that is with the same intensity all the way
- Interval training, that is with boosts of high intensity
- How was the intensity during the intervals?
- Much too high
- Too high
- Ok
- To low
- Much too low

How would you prefer to train?

- As I did before
- As I did in the study
- How did you experience training?
- What was good?
- What was bad?

1. Kendzierski D, DeCarlo KJ. Physical Activity Enjoyment Scale: Two Validation Studies. Journal of Sport & Exercise Psychology. 1991;13(1):50-64.
